# A large animal model of heritable pulmonary arterial hypertension using *BMPR2* gene–edited sheep

**DOI:** 10.1172/jci.insight.205583

**Published:** 2026-07-14

**Authors:** Sanjeev A. Datar, Nicholas Werry, Austin R. Brown, Devon S. Fitzpatrick, Oluwafemi Falade, Josephine F. Trott, Rachel Hutchings, Elena K. Amin, Jessica M. Morgan, Hythem Nawaytou, Gail H. Deutsch, Eric G. Johnson, Omar A. Gonzales Viera, Thomas F. Bishop, Tara Urbano Beach, Bret R. McNabb, Eric D. Austin, Jeffery R. Fineman, Alison L. Van Eenennaam

**Affiliations:** 1 Department of Pediatrics, UCSF, San Francisco, California, USA.; 2 Department of Animal Science, and; 3 School of Veterinary Medicine, UCD, Davis, California, USA.; 4 Department of Laboratory Medicine and Pathology, University of Washington, Seattle, Washington, USA.; 5 School of Medicine, Vanderbilt University, Nashville, Tennessee, USA.

**Keywords:** Cardiology, Pulmonology, Vascular biology, Cardiovascular disease, Molecular genetics

## Abstract

Pulmonary arterial hypertension (PAH) is a rare vascular disorder characterized by elevated pressure in pulmonary arteries, eventually leading to right ventricular failure. Approximately 50% of pediatric disease and 20% of adult disease can be linked to a genetic mutation, with nearly 70% of these cases involving mutations in the bone morphogenetic protein receptor type 2 (*BMPR2*) locus. Investigations using rodent models have made substantial advances in our understanding of *BMPR2* signaling; however, limited data exist regarding the onset and course of PAH, and etiologies for phenotypic expression in these patients remain unknown. In this work, we describe the development of an ovine model of heritable PAH. Because homozygous disruption of *BMPR2* is embryonic lethal, we developed heterozygous *BMPR2*-edited (*BMPR2^+/–^*) sheep by using a PAM-disrupting synonymous single-stranded oligodeoxyribonucleotide alongside a single guide RNA and Cas9-mediated gene editing strategy. The resulting *BMPR2^+/–^* lambs demonstrated cardiac and pulmonary vascular pathology that are consistent with *BMPR2* mutation–driven PAH observed in humans. Given the genetic and physiological similarities of *BMPR2^+/–^* sheep to humans with heritable PAH, this large animal model will serve as a vital platform for mechanistic molecular studies and will provide a much-needed preclinical model for extensive treatment evaluations.

## Abstract 2

We have created a large animal model of heritable pulmonary arterial hypertension, allowing for human-scale assessment of cardiopulmonary physiology and testing of promising new treatments.

## Introduction

Pulmonary hypertension (PH) represents a spectrum of disease processes whose underlying pathophysiology contributes to a common and often lethal phenotype: elevated pressure in the pulmonary arteries that eventually leads to right heart failure. Detailed investigations have been hampered by the lack of clinically relevant animal models of disease, and in particular, large animal models in which preclinical trials can be adequately performed. The seventh World Symposium on Pulmonary Hypertension classified pulmonary vascular disorders into 5 groups and 30 subgroups (1). Group 1 PH, or pulmonary arterial hypertension (PAH), is a rare but severe form of PH characterized by pulmonary vascular remodeling and increased pulmonary vascular resistance. Within Group 1, idiopathic PAH is the most common subgroup in adults from high-resource countries (2–4), whereas congenital heart disease–associated PAH is particularly important in pediatric populations. A much smaller subset of PAH is heritable, but over the past decade, the genetic basis underlying many cases of PAH has been increasingly defined. Mutations in 12 genes have now been strongly linked to PAH (5); indeed, between 70% and 80% of heritable PAH and 10% to 20% of idiopathic PAH can be attributed to mutations in known genes (6). In both children and adults, mutations in bone morphogenetic protein receptor type 2 (*BMPR2*) are the most common genetic cause of PAH (7). Patients with the *BMPR2* mutation are at high risk for developing PAH, but penetrance is highly variable, with pathologic expression occurring in approximately 42% of women but only approximately 14% of men (8, 9). Its onset can be insidious, despite careful monitoring and follow-up (10), and the natural history of this disease can be quite variable (11). Interestingly, a missense mutation in exon 3 of *BMPR2* is present among members of one of the largest familial cohorts of PAH (12, 13).

*BMPR2* encodes a type II serine/threonine kinase receptor in the TGF-β superfamily. A number of ligands signal through a transmembrane heterotetrameric complex composed of a BMPR2 dimer and 2 type 1 receptors and induce downstream activation of SMAD1/5/9 to regulate survival and propagation of vascular cells (14). Notably, in murine models, complete loss of *Bmpr2* is early embryonic lethal, failing to form organized structures or mesoderm (15), and *BMPR2* loss-of-function mutations have only been found in heterozygous individuals (16).

Sheep have long been regarded as high-fidelity models of newborn and infant physiology (17–23). As CRISPR/Cas9 gene editing has emerged as a routine methodology, genetic manipulation in sheep has now become logistically and economically feasible; indeed, a number of genetic ovine models of human disease have been recently described, including those for human deafness (24) and cystic fibrosis (25). Until now, however, a large animal model of hereditary PAH has not been successfully created, in part due to the challenges of introducing a heterozygous edit, thereby allowing the gene-edited fetuses to survive though gestation and parturition.

Many modern approaches to gene editing include the use of single-stranded oligodeoxyribonucleotides (ssODNs). These short DNA sequences serve as customizable donor templates for homology-directed repair (HDR), offering high editing efficiency and low incidence of off-target editing (26, 27). By combining CRISPR/Cas9-mediated cleavage and ssODN-mediated repair, it is possible to introduce a desired mutation to a specific site within the DNA (28). ssODNs have demonstrated remarkable efficiency in driving HDR in zygotes, making them particularly relevant for production of gene-edited animals (29). Importantly, an ssODN can be designed to replace the NGG protospacer–adjacent motif (PAM) with a silent PAM-disrupting sequence that does not alter the protein coded by that sequence, but that prevents all further CRISPR/Cas9 editing at that location (30).

Here, we have coupled a PAM-disrupting synonymous ssODN with single guide RNA–mediated (sgRNA-mediated) gene editing to produce a heterozygous *BMPR2* ovine model, providing an opportunity to study the molecular pathogenesis of PAH that is not currently possible in human patients or smaller mammals. The development of this model represents a substantial accomplishment in livestock gene editing, while the model itself provides a marked improvement in relevance to human physiology compared with murine studies, and will be an essential research tool for mechanistic understanding, strategies for disease management, and emerging therapeutics.

## Results

### HDR optimization.

Prior to embryo transfer, when electroporating 600 ng/μL ssODN, we found that sgRNA1 was significantly more efficient for HDR repair compared with sgRNA2 (*P* < 0.05). We found the editing efficiency of blastocysts when using the 600 ng/μL ssODN and sgRNA1 combination was 50% high (>66%), 35% intermediate (33%–66%), and 13.3% low (1%–33%) editing, with 1.7% being wild type (WT; <1% editing). Additionally, the HDR rate was 30% high (>20%), 21.7% intermediate (20%–5%), and 48.3% low to no (<5%) HDR. Based on these data, the combination of sgRNA1 with 600 ng/μL ssODN was used to generate heterozygous *BMPR2*-edited (*BMPR2^+/–^*) sheep, defined as sheep with at least 1 WT allele and at least 1 edited allele of *BMPR2*.

### Gene-edited lamb genotypes.

**Figure 1:**
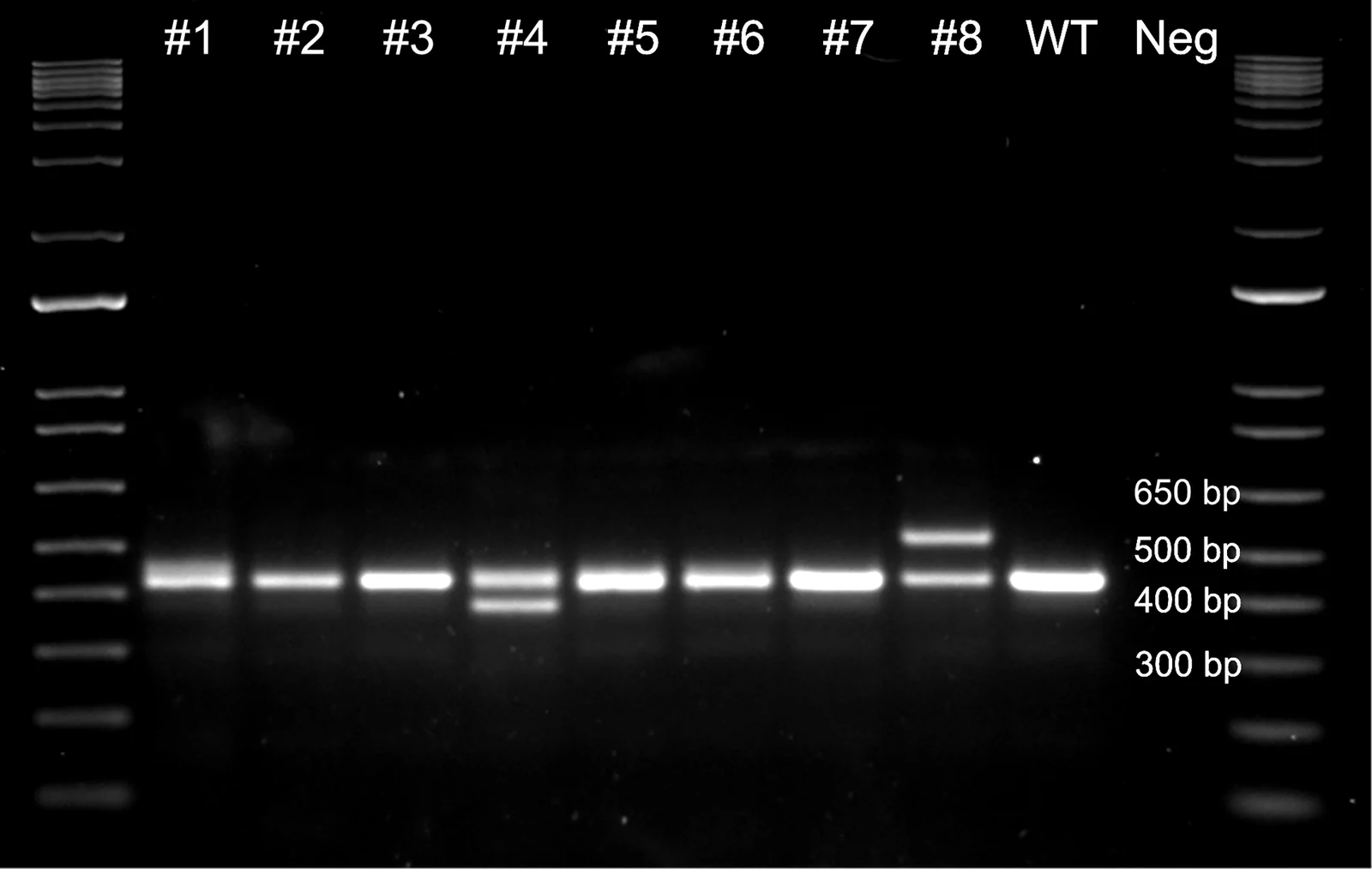
Agarose gel of *BMPR2* gene–edited lamb genotypes. A 431 bp WT amplicon of *BMPR2* was amplified by primers Intron2-F2 and Intron-3R and visualized on a 1.75% agarose gel to reveal obvious insertions and deletions in several of the 8 lambs. Neg, negative control.

**Figure 2:**
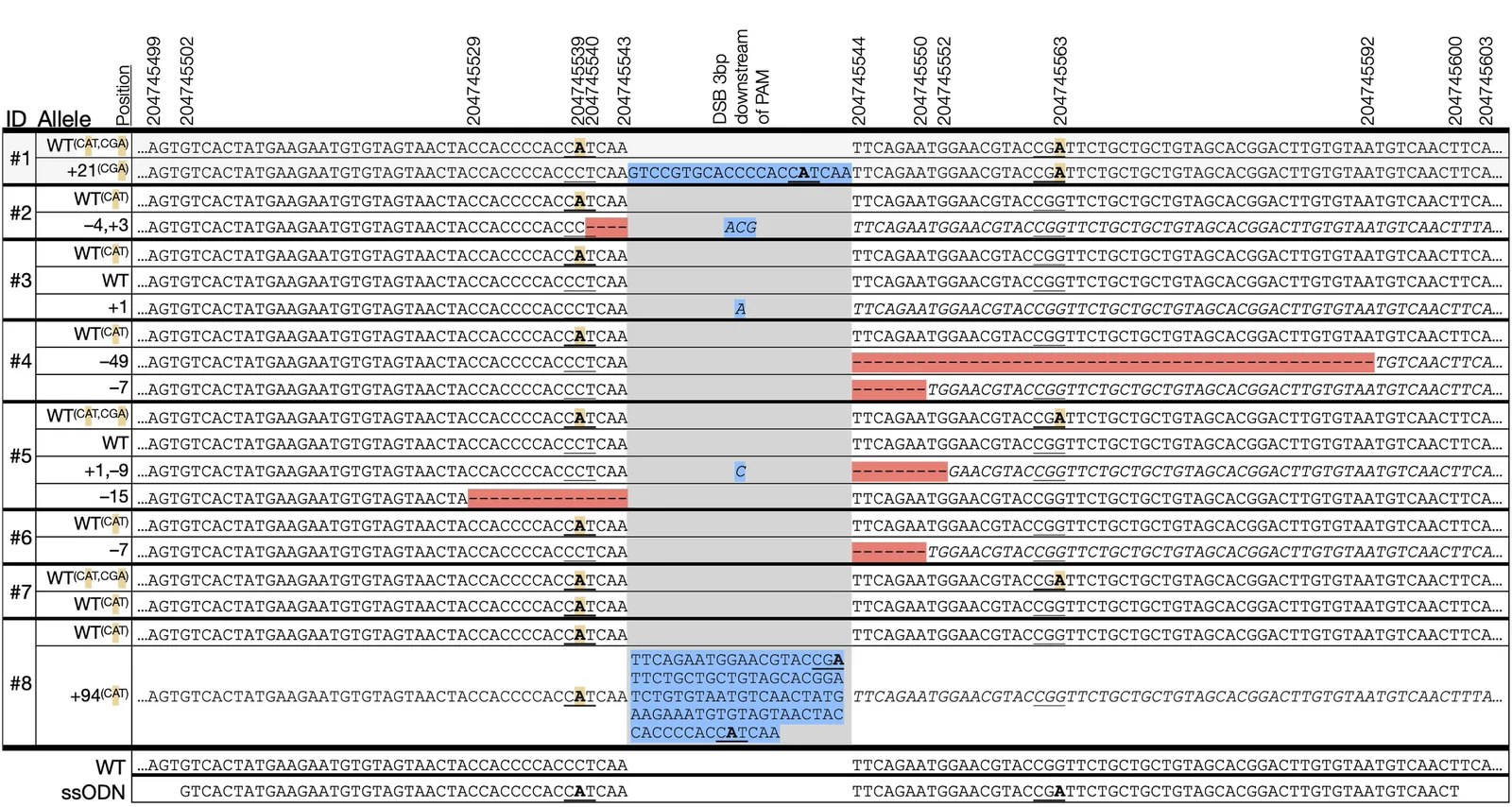
Edited *BMPR2* alleles identified by Amplicon-EZ sequencing. Alleles shown were observed in more than 1% of Illumina Amplicon-EZ reads. Positions are on *Ovis aries* Chr2 (NC_056055.1). Red highlighting indicates deleted sequence, blue indicates inserted sequence. Protospacer-adjacent motifs (PAMs) are underlined, with disrupting sequences bolded, and highlighted yellow when repaired using the single-stranded oligodeoxyribonucleotide (ssODN) template. The Cas9-induced double-stranded break 3 bp downstream of the PAM is identified in gray; resulting frame-shifted nucleotides are indicated in italics. The sgRNA1 PAM (CCT→CAT) was disrupted in at least 1 allele. The additional silent PAM disruption by the ssODN (CGG→CGA) at 204745563 was to disrupt the PAM for sgRNA2; the C/T variation at 204745602 is a known SNP (rs404303969). DSB, double stranded break; WT, wildtype.

Following electroporation and maturation, 5 to 6 blastocysts were surgically transferred to each of 8 surrogate ewes that responded to hormone synchronization, and pregnancies were confirmed by ELISA and an ultrasound at 6 weeks after transfer. A follow-up ultrasound at 9.5 weeks after transfer confirmed the viability of 8 fetuses past the period of embryonic lethality. Two ewes carried twins (nos. 2 and 3; 4 and 5) and 4 ewes carried single fetuses to term. Four lambs were live-born (3 female lambs nos. 5, 6, and 8; 1 male lamb no. 4), and 4 died perinatally. DNA was collected from all 8 lambs and the *BMPR2* region was amplified using Intron2-F2 and Intron3-R primers, revealing obvious insertions and deletions in exon 3 of *BMPR2* in 3 of the 8 lambs (Figure 1). The PCR products from each sheep were submitted for both Sanger sequencing and Illumina Amplicon-EZ sequencing to determine the alleles present and their relative abundance within each sample (Table 1), revealing at least 1 WT allele generated by HDR in all 8 lambs, and at least 1 edited allele in 7 lambs (Table 1 and Figure 2). The predicted amino acid sequences of the BMPR2 proteins from edited alleles are in Supplemental Table 1; supplemental material available online with this article; https://doi.org/10.1172/jci.insight.205583DS1 Seven of the 9 edited alleles are expected to result in dramatically truncated BMPR2 proteins, including both the 49 bp and 7 bp deletion alleles of the live *BMPR^+/–^* male lamb no. 4. All live-born lambs each carried at least 1 *BMPR2*-knockout allele (Table 1 and Supplemental Table 1).

### Expression analysis.

**Figure 3:**
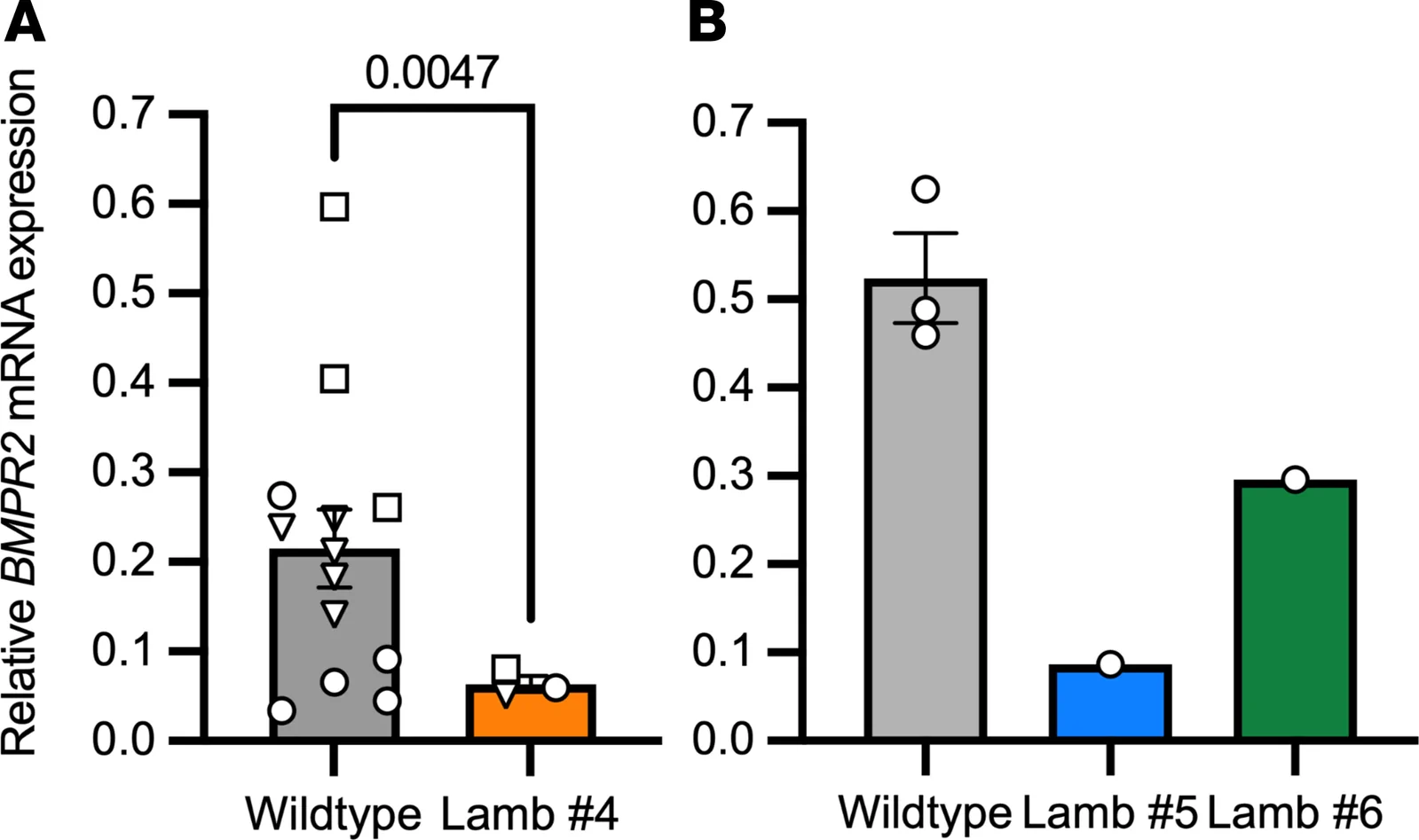
Relative *BMPR2* mRNA expression in WT and *BMPR2*-edited F0 lambs quantified by qPCR. (**A**) Expression analysis of live-born lamb no. 4 ear (square), heart (triangle), and lung (circle) samples compared with WT controls (*n* = 3–5). Lamb 4 was compared to WT using an unpaired, 2-tailed Welch’s *t* test. (**B**) Expression analysis of live-born lamb 5 and 6 lung samples compared with WT controls (*n* = 3). Mean and SEM values are shown with individual data points overlaid.

**Figure 4:**
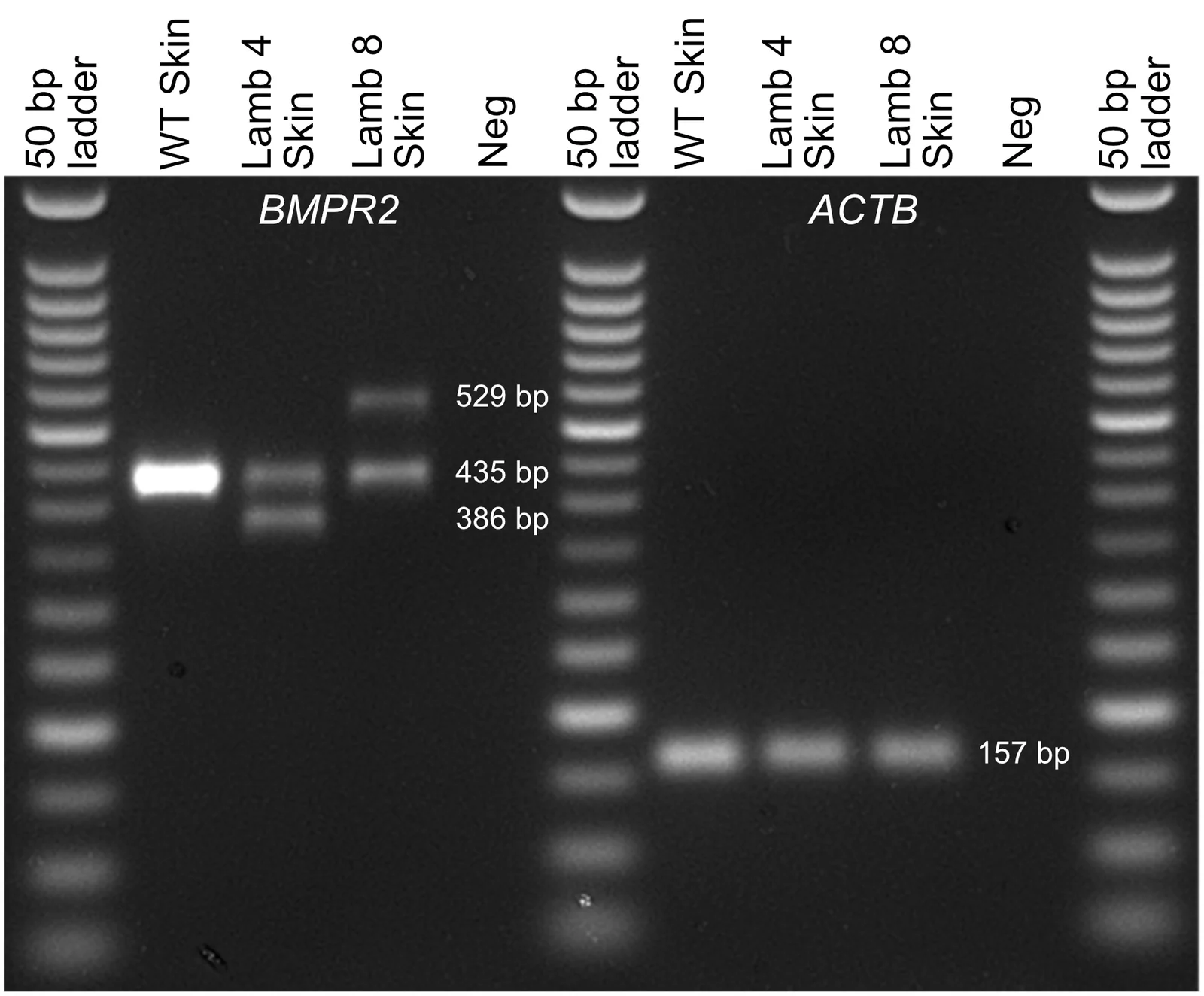
Expression of *BMPR2* transcripts in ear skin of WT and *BMPR2*-edited F0 lambs 4 and 8. The WT *BMPR2* allele (435 bp), 49 bp deletion allele (386 bp), and 94 bp insertion allele (529 bp) are easily visualized. *ACTB* expression (157 bp) was used as a housekeeping control.

To evaluate the impact of *BMPR2* gene editing on transcript expression, we quantified WT *BMPR2* mRNA levels by quantitative (qPCR) in either lung or lung, heart, and ear skin from live-born F0 lambs and normalized expression to the reference gene *ACTB* (Figure 3). *BMPR2* mRNA levels found in lung, heart, and ear skin were reduced significantly in lamb no. 4 relative to WT (*P* = 0.003; Figure 3A). *BMPR2* mRNA levels in lung tissues were markedly reduced in lamb no. 5 relative to WT, but the reduction was less in lamb no. 6 (Figure 3B). The 94 bp insertion in exon 3 of *BMPR2* in lamb no. 8 reconstituted the *BMPR2* WT sequence such that our qPCR assay for WT *BMPR2* mRNA could not be used for this allele. We therefore extracted RNA from ear skin of lambs nos. 4, 8, and a WT control and did semiquantitative reverse transcription PCR (RT-PCR) to visually assess the relative levels of their *BMPR2* transcripts (Figure 4). The WT allele was visible in all 3 lambs along with the 49 bp deletion allele in lamb no. 4, which had approximately the same intensity as the lamb no. 4 WT allele. The 7 bp deletion allele in lamb no. 4 was indistinguishable from the WT allele. The 94 bp insertion allele in lamb no. 8 appeared less intense than the lamb no. 8 WT allele (Figure 4).

### Cardiac evaluations.

**Figure 5:**
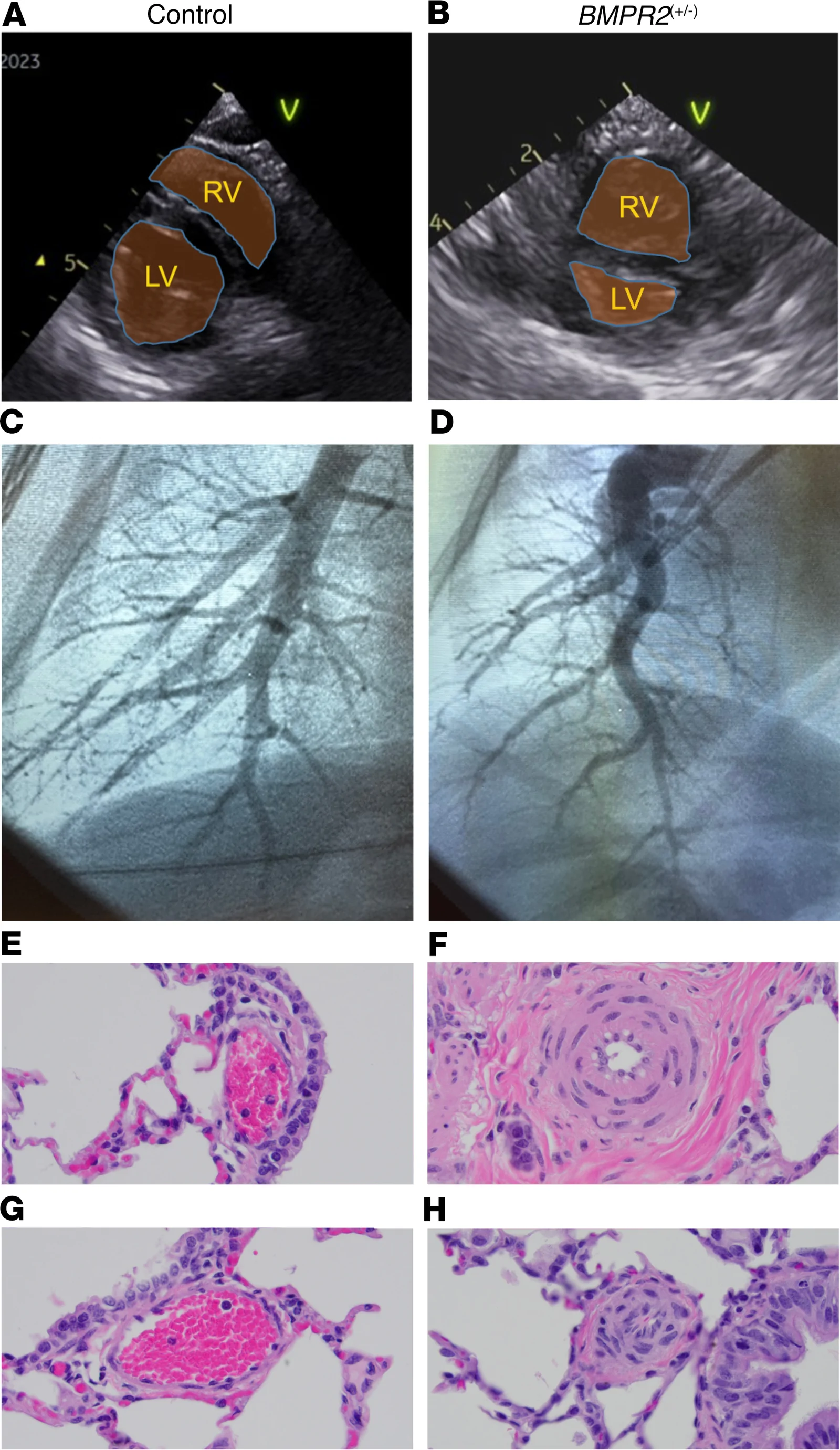
Pulmonary hypertension evaluation. Echocardiography at 3 weeks of age, with representative images of (**A**) control versus (**B**) *BMPR2^+/–^* female (no. 8). RV, right ventricle; LV, left ventricle; V, ventral. Right pulmonary angiography of (**C**) control and (**D**) *BMPR2^+/–^* lamb no. 8. H&E staining (original magnification, ×60) of pulmonary arterioles from *BMPR2^+/–^* lambs no. 5 (**F**) and 6 (**H**) associated with medium-sized airways. (**E** and **G**) Arterioles from age-matched control lambs.

Of the 4 live-born gene-edited lambs, 3 were female. At 3 weeks of age, echocardiograms were performed on all 4 *BMPR2^+/–^* lambs. Compared with control (Figure 5A), in 2 of the 3 female *BMPR2^+/–^* lambs (nos. 6 and 8), there was evidence of end systolic septal flattening and right ventricular (RV) hypertrophy, with an elevated left ventricular (LV) eccentricity index of 1.6 and 1.5, respectively (Table 2). An LV eccentricity index above 1.1 is considered abnormal, with a value above 1.3 highly suggestive of PH. The echocardiogram and LV eccentricity index of the male (no. 4) and the third female (no. 5) *BMPR2^+/–^* lambs were normal. Interestingly, a ratio of LV to RV end diastolic dimension (LVEDD/RVEDD) of 1 indicated that lamb no. 8 had evidence of RV dilation, while lamb no. 6, with a preserved LVEDD/RVEDD ratio, did not (Table 2).

Each of the 3 female *BMPR2^+/–^* female lambs were noted to have a persistently patent ductus arteriosus (PDA) at 7 to 8 weeks of age. In contrast, a PDA was not observed in any WT lambs (*n* = 6) at 4 weeks of age by computed tomography angiography (data not shown).

At 11 weeks of age, we performed a right heart catheterization in *BMPR2^+/–^* ewe lamb no. 8, and an age-matched control (Table 3). We did not find a PDA in either lamb. In 21% FiO_2_, the *BMPR2^+/–^* lamb’s cardiac index was 4.1 L/min/m^2^, the mean pulmonary artery pressure was 29 mmHg, and the calculated pulmonary vascular resistance (PVR) was 6.3 indexed Wood units (iWU). In response to acute vasodilator testing (100% O_2_ + 40 ppm inhaled nitric oxide), *BMPR2^+/–^* lamb no. 8 had a 41% decrease in mean pulmonary artery pressure, and the calculated PVR was 3 iWU, a 52% decrease from baseline conditions. Compared with a normal pulmonary artery morphology in the control lamb (Figure 5C and Supplemental Video 1), right pulmonary angiography demonstrated a tortuous and pruned vasculature in the *BMPR2^+/–^* lamb that is characteristic of PAH (Figure 5D and Supplemental Video 2).

### Lung histopathology.

**Figure 6:**
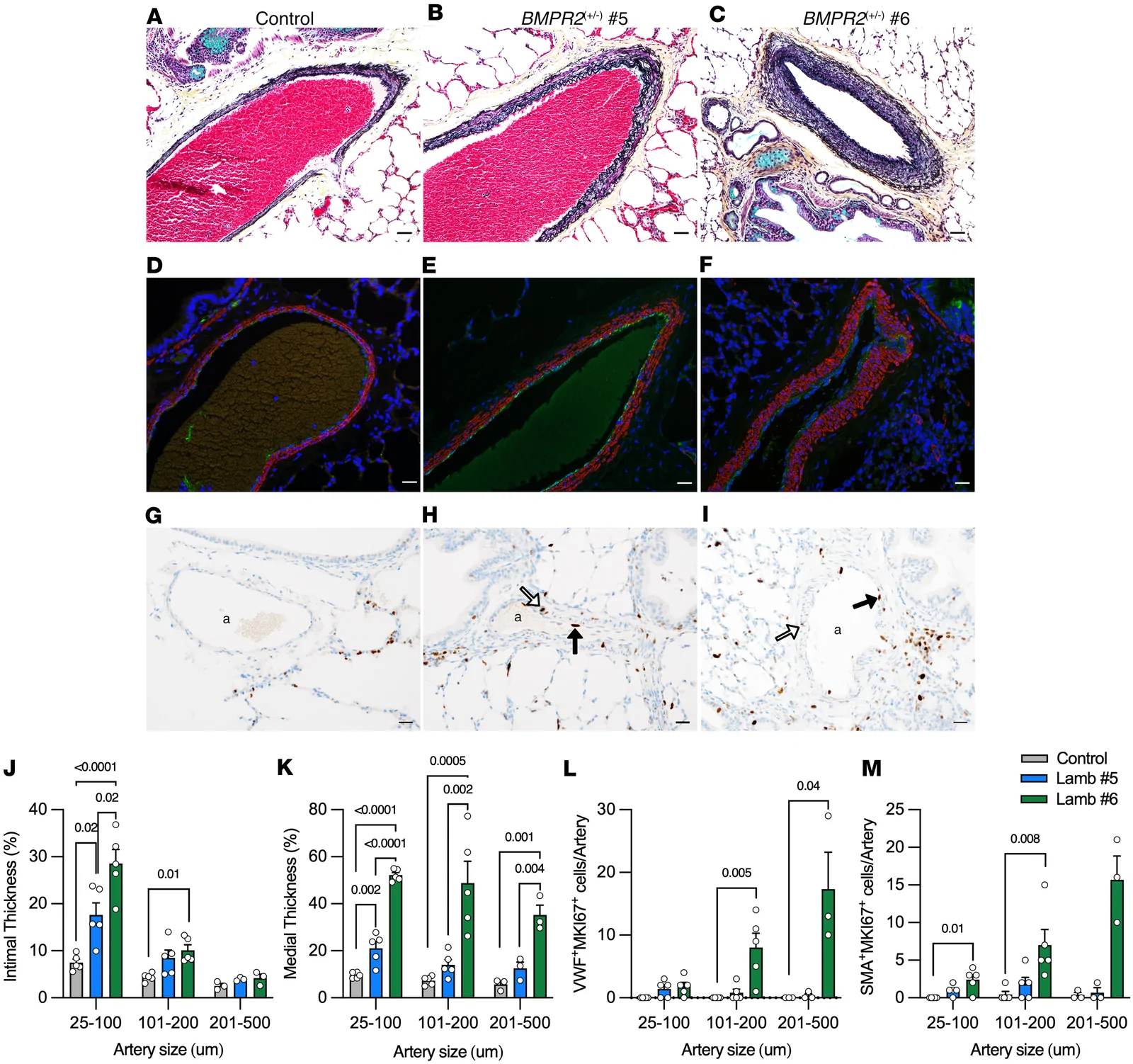
Histopathology of pulmonary arteries from lungs from *BMPR2^+/–^* lambs 5 and 6 compared with control. (**A**–**C**) Pentachrome staining identifies arterial wall hypertrophy in *BMPR2^+/–^* lambs. Scale bars: 50 μm. (**D**–**F**) Immunofluorescent staining highlights expansion of both endothelial (von Willebrand factor [VWF], green) and smooth muscle (α-smooth muscle actin [SMA], red) layers. Scale bars: 25 μm. (**G**–**I**) MKI67 staining: Open arrowheads mark MKI67^+^ proliferating cells in the smooth muscle layer and the closed arrowheads mark MKI67^+^ proliferating cells in the endothelium. a, pulmonary artery. Scale bars: 25 μm. (**J**–**M**) For morphometry, 5 vessels each were analyzed for the small- and medium-sized arteries in each animal, with 3 each for the large-sized arteries. Differences in intimal and medial thickness (**J** and **K**) within each arterial size range were determined using 1-way ANOVA with Tukey’s correction for multiple comparisons. For endothelial (**L**) and smooth muscle cell (**M**) proliferation, Kruskal-Wallis non-parametric tests were used to compare lambs. Mean and SEM values are shown with individual data points overlaid.

Two of the female F0 *BMPR2^+/–^* lambs (nos. 5 and 6) were euthanized at 8 and 6 weeks of age, respectively. Lamb no. 5 developed sepsis that was non-responsive to treatment; lamb no. 6 was born with arthrogryposis of the right forelimb as well as mandibular brachygnathia (Supplemental Figure 1), and she was unable to bear weight on her front leg and thus unable to stand or ambulate due to osteomyelitis and pathologic fractures of the right distal phalanx. Postmortem lung samples for each were processed and compared to an age-matched control. In both euthanized *BMPR2^+/–^* lambs (Figure 5F, no. 5 and Figure 5H, no. 6), there was prominent medial hypertrophy with focal luminal obliteration of pulmonary arteries associated with medium-sized airways compared with age-matched controls (Figure 5, E and G). Pentachrome staining highlighted the pulmonary arterial remodeling in the female F0 *BMPR2^+/–^* lambs (Figure 6, B and C), which affected approximately 75% of the vessels evaluated in lamb no. 5 and all vessels in lamb no. 6 (data not shown). Analysis of von Willebrand Factor (VWF) and α-smooth muscle actin (SMA) demonstrated that the small-sized pulmonary arteries in each heterozygous lamb had at least a 2.2-fold increase in intimal (Figure 6J) and medial wall (Figure 6K) thickness (*P* < 0.05). Interestingly, lamb no. 6 had 2.5- to 3.5-fold more muscularization at all vessel sizes compared with lamb no. 5 (Figure 6K; *P* < 0.05), consistent with its more abnormal eccentricity index (Table 2). Furthermore, MKI67 staining identified proliferating arterial endothelial (closed arrowheads) and smooth muscle cells (open arrowheads) only in the lungs from *BMPR2^+/–^* lambs (Figure 6, H and I). Compared with the control, lamb no. 6 also had significantly more intimal hyperplasia in the medium- and large-sized arteries (Figure 6L; *P* < 0.05), with significantly more smooth muscle cell proliferation in the small- and medium-sized arteries (Figure 6M; *P* < 0.05). Interestingly, no TUNEL-positive cells were observed in any of the arteries assessed, with exception of rare positive cells in the adventitia (data not shown).

### Germline transmission of mutant allele.

Illumina Amplicon-EZ sequencing of sperm collected from the *BMPR2^+/–^* ram lamb no. 4 revealed a higher relative proportion of a secondary knockout allele in his germline compared with his blood; the 7 bp deletion represented 17% of sperm reads, while the 49 bp deletion was consistent at 58% (Table 1).

Blastocysts created by in vitro fertilization using semen collected from *BMPR2^+/–^* ram lamb no. 4 were 47.8% (11/23) WT/–49 bp genotype, 17.4% (4/23) WT/–7 bp genotype, and 34.7% (8/23) were WT/WT^CAT^, with one allele having been generated by HDR repair with the telltale silent “CAT” PAM-disrupting sequence at the sgRNA1 PAM site (Supplemental Figure 2).

## Discussion

### Heterozygous BMPR2-knockout model generation.

We have successfully developed a heritable large animal model of PAH. An ssODN was used to produce *BMPR2^+/–^* sheep by introducing a silent mutation at the PAM site to prevent further editing in 1 allele while the second allele was disrupted by NHEJ-induced indels. Heterozygous editing was essential to avoid the embryonic lethality associated with complete loss of BMPR2 (15). To our knowledge, this is the first documentation of live-born sheep generated with an intentional CRISPR/Cas9-induced heterozygous edit, resulting in 4 live-born gene-edited lambs, each uniquely heterozygous for *BMPR2*.

**Figure 7:**
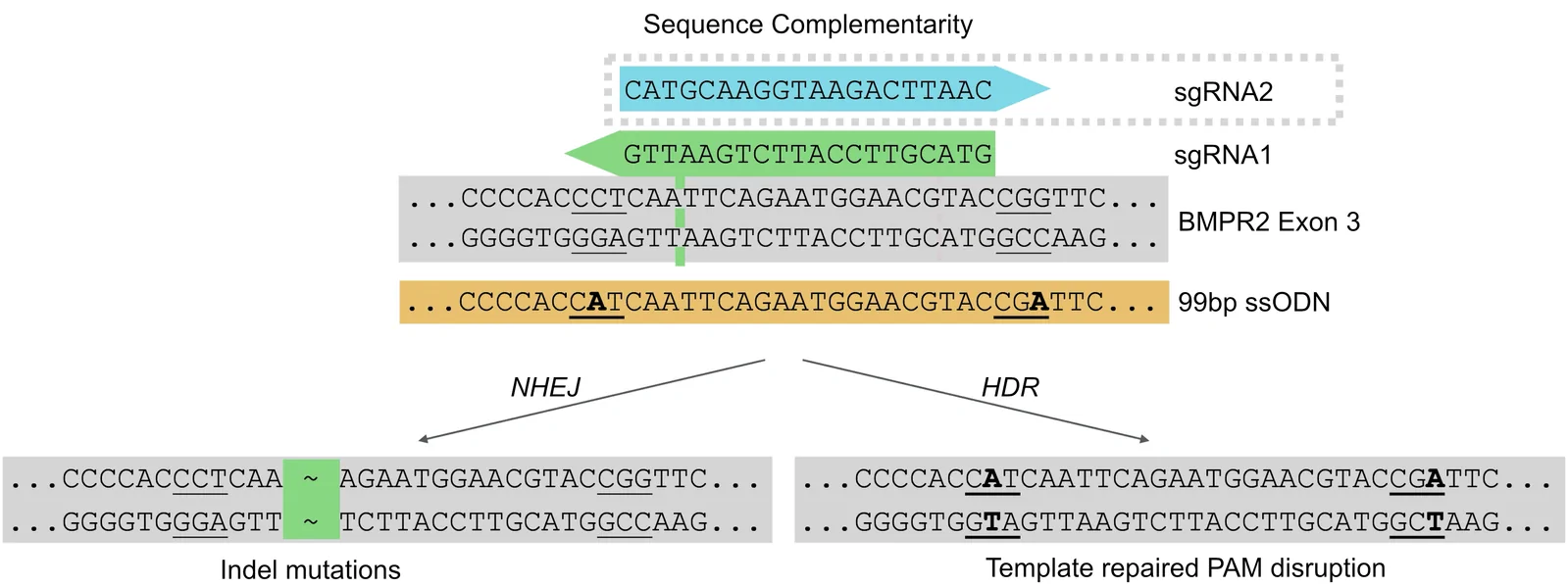
Editing sequence design. Single guide RNA 1 (sgRNA1) (green) and sgRNA2 (blue) were targeted to exon 3 of *BMPR2*. sgRNA1 was used in production of gene-edited lambs, and sgRNA2 was used solely for in vitro testing (indicated by dotted box). Protospacer-adjacent motifs (PAMs) are indicated by underlining; mismatches relative to the Oar_v3.1 reference genome are indicated in bold. The location where sgRNA1 would be predicted to introduce a double-stranded break (DSB) is indicated by the green dashed line. Non-homologous end joining (NHEJ) repair of the DSB will repeatedly cleave and introduce indel mutations until the guide sequence is disrupted, while homology-directed repair (HDR) using the 99 bp single-stranded oligodeoxyribonucleotide (ssODN) will introduce a synonymous silent mutation, disrupting the PAM of sgRNA1 (CTT→CAT) and preventing further Cas9 activity. The additional PAM-disrupting mutation (CGG→CGA) towards the 3′ end of the ssODN was designed to disrupt the PAM of sgRNA2.

During CRISPR/Cas9-mediated genome editing, the process of HDR using an ssODN repair template is referred to as single-stranded template repair and results in higher genome editing efficiencies than HDR pathways that use dsDNA repair templates. It has been observed that knockin rates are higher when the ssODN is complementary to the PAM-containing (non-target) strand (31). This has been linked to the direction of DNA resection after Cas9 cleavage. We found that knockin rates were higher with sgRNA1 where the 99 bp ssODN was complementary to the PAM-containing strand than with sgRNA2, which targeted the other strand (Figure 7). In this experiment, all 8 of the gene-edited lambs showed evidence that at least 1 allele was repaired using the ssODN template (Table 1). Each contained the synonymous PAM-disrupting sequence (CAT) targeted in this experiment (NC_056055.1:g204745539), indicating successful use of the ssODN template. However, the silent substitution on the ssODN (CGA) (NC_056055.1:g204745563), located downstream of the exon 3 cut site, enabled us to determine that the entirety of the 99 bp ssODN was only used in the HDR repair of 1 WT allele in 3 lambs (nos. 1, 5, and 7). It was also notable that in 2 progeny, nos. 1 and 8, which incorporated 21 bp and 94 bp insertions, respectively, the ssODN template was incorporated into the insertion, as evidenced by the sequence of the PAM-disrupting mutations (Figure 2).

In instances where the double-stranded break was repaired by NHEJ, variations in indel mutations led to the generation of different knockout alleles in most animals. In some instances, multiple alleles were present, suggesting that some editing occurred after the 1-cell zygote stage. In the male *BMPR2^+/–^* lamb (no. 4), a third allele was present at a higher relative proportion in the germline compared with blood, meaning that he would be expected to produce approximately 2 out of 3 *BMPR2^+/–^* and 1 out of 3 *BMPR2^+/+^* offspring versus the typical 1:1 ratio. This 2 to 1 heterozygous/WT *BMPR2* ratio was confirmed by germline testing of his sperm (Supplemental Figure 2).

Each of the 8 gene-edited lambs that developed to term had at least 1 WT allele, supporting the idea that loss of both *BMPR2* alleles is embryonic lethal. The 4 lambs that died perinatally were after term and appeared to have died of complications of failed labor associated with large offspring syndrome, a known complication of in vitro fertilization in sheep (32).

### BMPR2-knockout model phenotypic analyses.

By 2 months of age, each of the 3 F0 *BMPR2^+/–^* female lambs demonstrated evidence of pulmonary vascular disease. Consistent with the variable penetrance and expressivity seen in patients that carry a *BMPR2* mutation, there was a gradation of disease severity. One *BMPR2^+/–^* female (no. 8) exhibited a severe PH phenotype by 5 weeks of age. Her echocardiogram showed a globular hypertrophic right ventricle, septal flattening, an abnormally elevated eccentricity index, and an LVEDD/RVEDD ratio consistent with RV dilation in the setting of elevated RV pressure (Figure 5B and Table 2). A subsequent right heart catheterization at 11 weeks of age confirmed demonstrable PAH; she had an elevated mean pulmonary artery pressure greater than 20 mmHg (29 mmHg in 21% FiO_2_), with a normal pulmonary arterial wedge pressure and an elevated PVR of 6.3 iWU (Table 3), as well as a tortuous right pulmonary artery by angiography with evidence of distal arterial pruning (Figure 5D).

Each of the 2 F0 *BMPR2^+/–^* females that were euthanized showed histopathologic evidence of pulmonary arterial remodeling, with hyperproliferation and hypertrophy of the intimal and medial layers of small- and medium-sized arteries (Figure 6), and evidence of luminal obliteration (Figure 5, F and H). Importantly, although lamb no. 6 had an abnormal eccentricity index, the LVEDD/RVEDD ratio was normal (Table 2), suggesting elevated RV pressures but with preserved RV dimensions. *BMPR2^+/–^* ewe lamb no. 5 had a normal echocardiogram with a normal calculated eccentricity index (Table 2), suggesting a disparity between histopathology and echocardiographic evidence of PAH.

Of note, lamb no. 5 developed sepsis and was euthanized at 8 weeks of age. We believe her infection was unlikely related to her *BMPR2* mutation. However, lamb no. 6 was born with arthrogryposis of the right forelimb as well as mandibular brachygnathia (Supplemental Figure 1). She developed osteomyelitis and pathologic fractures of the right distal phalanx and was euthanized at 6 weeks of age. We do not know whether the BMPR2 mutation impacted her skeletal phenotype, but given the important role of bone morphogenetic protein signaling in skeletal patterning and limb development (33), this warrants further investigation.

Interestingly, the male *BMPR2^+/–^* lamb (no. 4) had normal echocardiograms and hemodynamics and was clinically asymptomatic with a histologically normal pulmonary vasculature (data not shown). Critically, 2 mutant *BMPR2* alleles are represented in his germline (Table 1), and these sperm are functional and can produce viable blastocysts bearing WT, –49, or –7 bp alleles (Supplemental Figure 2), which suggests that, if bred to WT ewes, this *BMPR2* heterozygous ram should be able to sire *BMPR2^+/–^* and WT F1 sibling offspring for sustained future studies.

The observation that the 3 female *BMPR2* heterozygous lambs had a PDA is intriguing. To our knowledge, PDA has not been described as a phenotype in rodent investigations of *Bmpr2* mutations (34–38). Of note, we have never previously observed a PDA in any WT lamb that we evaluated. *BMPR2* mutations may be associated with congenital heart defects that include PDA (39), but it is not at all clear that *BMPR2* mutations play a causal role in the development of PDA or other congenital heart disease. Similarly, it is unknown whether delayed closure of the ductus arteriosus is a marker of the severity of pulmonary vascular disease in these female *BMPR2^+/–^* lambs, or might be contributing to their pathophysiology, and warrants further study.

In summary, this large animal model of heritable PAH offers human-scale assessment of cardiopulmonary physiology, such as the evaluation of right ventricle–pulmonary artery coupling, proximal pulmonary artery mechanics, and pulmonary compliance. In addition, it will allow meaningful investigation into the effects of normal development on disease progression, particularly with respect to the transition to sexual maturity and exposure to clinically relevant “second hits” like pregnancy (40, 41), high altitude (40), and congenital heart disease (42, 43). Lastly, it may allow for preclinical device and drug delivery studies, biomarker discovery, and testing of promising therapeutics, including gene therapies that will lead to improved quality of life for patients affected by PAH.

## Methods

### Sex as a biological variable.

Our study evaluated both female and male F0 animals, and our findings support what has been observed in humans: variable penetrance of phenotype that is expressed more frequently in females.

### Editing construct design.

An sgRNA CRISPR/Cas9 approach to target exon 3 of the ovine *BMPR2* gene, in combination with an ssODN, PAM-disrupting, synonymous HDR template was employed to generate *BMPR2^+/–^* sheep (Figure 7). Two sgRNAs (sgRNA1 and -2) targeting *BMPR2* exon 3 on opposite strands were designed using *Ovis aries* reference genome Oar_v3.1 (RefSeq GCF_000298735.1) in ChopChop (44), while the 99 bp ssODN was manually designed to silently disrupt the NGG PAM of both guides by inducing a G→T mutation at 1 of the 2 Gs in each NGG PAM sequence (Table 4).

### Presumptive zygote production.

Ovine ovaries were collected fresh from a local abattoir (Superior Farms), stored in 37°C saline, and aspirated within 2 hours of collection. Cumulous oocyte complexes (COCs) were collected with a 21-G needle, those with several layers of cumulous cells were matured in batches of 50 for 22–24 hours in 400 μL of maturation media (Stroebech IVM for Small Ruminant Oocytes, PETS) in a humidified incubator at 38.5°C, 5% CO_2_.

Following maturation, COC batches were rinsed 3 times in in vitro fertilization (IVF) media and placed in 400 μL IVF media (Stroebech IVF Media for Small Ruminant, PETS). Frozen semen straws, from a Hampshire ram, were thawed in 37°C water for 1 minute prior to washing twice in 1 mL semen preparation media (Stroebech Semen Wash for Small Ruminants, PETS), centrifuging at 300*g* for 5 minutes. Fertilization media was used to adjust sperm concentration to 2 × 10^6^ sperm/mL, 50 μL of which was used to fertilize each batch for 6 hours in a humidified incubator at 38.5°C, 5% CO_2_.

### Electroporation and embryo culture.

Presumptive zygotes 6 hours after fertilization were denuded by 3 minutes of vortexing in synthetic oviductal fluid–HEPES (SOF-HEPES) (Supplemental Table 2), then washed 3 times in SOF-HEPES and 3 times in Opti-MEM (Thermo Fisher Scientific). Zygotes were moved into a fresh drop of Opti-MEM and mixed with the ssODN alongside ribonucleoprotein complexes comprised of 100 ng/μL sgRNA1 or -2, 200 ng/μL Cas9, and 600 ng/μL ssODN in a 20 μL final volume. The mixture was loaded into a 1 mm Electroporation Cuvette (Bulldog-Bio) and electroporated via the NEPA21 Super Electroporator using poring conditions of 2 bipolar pulses of 30 volts for 3.5 msec and transfer conditions of 50 msec each. Recovered presumptive zygotes were washed 3 times in SOF-HEPES and groups of 50 were placed in 500 μL of in vitro culture media (Stroebech IVC Media for Small Ruminants, PETS) covered with 400 μL mineral oil (Ovoil, Vitrolife). Embryos were cultured in IVC media for 7 days in a humidified incubator at 38.5°C, in 5% CO_2_, 5% O_2_, and 90% N_2_.

### PCR analyses.

For brevity, conditions (primer sequences, reagent composition, thermocycling conditions) for all PCR assays in subsequent sections are presented in Supplemental Table 3.

### Genomic analyses of blastocysts.

At 7 days after fertilization, all zygotes that had advanced to the blastocyst stage of development (except for those to be used for embryo transfer) were collected in 10 μL QuickExtract DNA Extraction Solution (Biosearch Technologies), and incubated at 65°C for 6 minutes, 98°C for 2 minutes, then cooled to 4°C to collect DNA. PCR products were gel purified prior to submission for Sanger sequencing (Genewiz). Sequences were analyzed using TIDER (45), a computer model that quantifies the frequency of targeted small nucleotide changes introduced by CRISPR in combination with HDR using a donor template.

### Embryo transfer.

Eazi Breed CIDRs Progesterone inserts (Zoetis) were placed for 6 days to synchronize estrus in multiparous surrogate ewes. On the day of insert removal, intramuscular injections of prostaglandin F2-α analog (10 mg dinoprost tromethamine; Zoetis) and PG600 (400 IU PMSG, 200 IU hCG; Intervet) were given. Ewes were food restricted for 24 hours and water restricted for 12 hours before undergoing a minimally invasive laparoscopic procedure, as previously described (46). Briefly, ewes were sedated by intravenous injection of 1.1–2.2 mg/kg of ketamine and 0.2–0.3 mg/kg of Midazolam prior to surgery. Lidocaine (2%) was used as a local anesthetic at the incision site. Ewes were placed in the Trendelenburg position and had their abdomen insufflated with CO_2_. The ovaries and uterus were visualized via 0-degree laparoscope in the caudal abdomen, and 5–6 blastocysts were transferred into the uterine horn ipsilateral to the corpus luteum. Surplus blastocysts were analyzed for editing efficiency as described above.

### Genotype analysis of F0 lambs.

Genomic DNA was extracted from either tissue from stillborn lambs or blood from live-born lambs using the DNeasy Blood and Tissue Kit (Qiagen). PCR products were gel purified (Figure 1), including 2 bands extracted independently for both lambs, no. 4 and no. 8, and subjected to Sanger sequencing (Genewiz). For quantification of alleles and identification of rare alleles in mosaic lambs, DNA was PCR amplified, all products 300–550 bp were gel purified as a single sample per lamb, and subjected to Illumina Amplicon-EZ next-generation sequencing (Genewiz).

### RNA extraction and RT-PCR.

For WT controls, ear, lung and heart tissues were collected under hygienic conditions from sheep immediately following slaughter at an abattoir, snap frozen in liquid nitrogen, and stored at –80°C until processing. Ear tissues from lambs 4 and 8 were collected by punching the ear of the living animal while lung tissues from lambs 5 and 6 were collected immediately following euthanasia, snap frozen with liquid nitrogen, and stored at –80°C. Total RNA was extracted by homogenizing in QIAzol reagent (Qiagen) following the manufacturer’s instructions. RNA concentration and purity were determined using a NanoDrop 1000 (Thermo Fisher Scientific). RNA integrity was verified by confirming presence of non-degraded *28S* and *18S* bands on a MOPS-formaldehyde 1.2% agarose gel electrophoresis. Total RNA was treated with TURBO DNase (Invitrogen) according to the manufacturer’s instructions. For cDNA synthesis, 1 μg of total RNA was reverse transcribed using GoScript Reverse Transcriptase (Promega) in 20-μL reactions according to the manufacturer’s instructions with both Oligo(dT) (Promega) and 50 ng of random hexamers. Each RT reaction was accompanied by a no-RT control in which the reverse transcriptase was replaced by RNase-free water, and an absence of contaminating DNA was validated by PCR. cDNA was diluted with an equal volume of RNase-free water and stored at –80°C.

Expression of *BMPR2* alleles in ear skin tissue from a WT sheep, and from lambs 4 and 8 was examined by semiquantitative PCR on cDNA. The resulting PCR products were run on a 2% agarose gel where the size and intensity of the bands were estimated.

### qPCR.

β-Actin (*ACTB*) was used as housekeeping control and primers for *BMPR2* and *ACTB* TaqMan quantitative PCR (qPCR) reactions were designed to span exon-exon junctions, when possible, to avoid genomic DNA amplification (Supplemental Table 3). Locking nucleic acid probes for WT *BMPR2* that included either the WT CCT sequence or the sgRNA1 PAM-disrupting “CAT” sequence encoded in the ssODN (Figure 7) were designed using OligoArchitect Online (Sigma-Aldrich). Probes were labeled 3′ with black hole quencher-1 (BHQ-1) and 5′ with either 6-carboxyfluorescein (6-FAM for *ACTB*) or SUN (for *BMPR2*; Integrated DNA Technologies). Each PCR run included a no-template control containing all reagents except cDNA. All samples, standards, and controls were assayed in duplicate on each plate.

The levels of *BMPR2* and *ACTB* mRNA expression were determined using a relative standard curve prepared from 6 four-fold serial dilutions of a lung RNA sample known to have high levels of *BMPR2* gene expression. A standard curve was used on every qPCR plate. All standard curves had a linear regression coefficient of determination of at least 97 and *ACTB*’s efficiency was 86%, while *BMPR2*’s was 77%. Standard curves were generated by linear regression using Ct versus log(dilution factor). The *BMPR2* and *ACTB* levels in each sample were calculated from Ct values using the standard curve. Data were expressed as the ratio between *BMPR2* and *ACTB* expression levels, yielding a normalized relative expression level of *BMPR2* mRNA. The results presented are the average across 2 assays.

### Germline transmission by BMPR2^+/–^ F0 ram.

Semen was collected from the gene-edited ram (no. 4) following sexual maturity. DNA was extracted from the semen and *BMPR2* was PCR amplified and submitted for Illumina Amplicon-EZ next-generation sequencing (Genewiz). The semen was also used to fertilize sheep oocytes in vitro as described above. The resulting blastocysts were analyzed as described above in *Genomic analyses of blastocysts* and PCR products were sequenced using Sanger sequencing to identify which allele was transmitted.

### Echocardiograms of BMPR2^+/–^ F0 lambs.

Transthoracic echocardiograms were performed on non-sedated 3-week-old lambs by a single echocardiographer. The coat was clipped bilaterally and prepared with alcohol and ultrasound gel. Cine-loops were obtained with an ultrasound machine (Vivid IQ Premium V204; GE Healthcare) equipped with a 6S-RS Sector Transducer and simultaneous ECG recording. Standard 2-dimensional views obtained from the right chest wall included 4-chamber apical view, long axis views of the RV and LV outflow tracts, and short axis views at the aortic, mitral, and chordal level. M-mode images of the aortic valve, mitral valve, and left ventricle at the chordal level were also obtained. From the left chest wall, long axis 2-dimensional images of the left atrium, aortic valve, and pulmonic valve were obtained. A second echocardiographer, who was blinded to the animal identification, evaluated the images in Syngo (Siemens Healthineers, Version VA40D), and using the parasternal short axis views in systole, calculated the eccentricity index as previously described (47).

### Right heart catheterization of BMPR2^+/–^ F0 lamb.

At 11 weeks of age, 1 female gene-edited lamb (no. 8) and an age-matched control underwent hemodynamic right heart catheterization via percutaneous access of the right internal jugular vein, using standard evaluation techniques for pediatric PH (48). Lambs were maintained in a supine position under inhaled anesthesia with 1%–3% isoflurane and were mechanically ventilated using a GE Healthcare Datex Ohmeda Aestiva5 ventilator. End-tidal CO_2_ was maintained between 35 and 45 mmHg, with confirmation as needed by arterial blood gases (ABL 90 Flex; Radiometer America) from a peripheral arterial line. Maintenance fluids (Lactated Ringers) were delivered intravenously during the procedure. Percutaneous internal jugular vein 5 Fr or 6 Fr introducer sheaths were placed, and lambs were heparinized with 150–300 IU/kg heparin sodium. Activated clotting times (ACTs) were monitored to maintain adequate anti-coagulation (ACT monitor; Actalyke Mini-II; Helena Laboratories) during the study.

Baseline hemodynamics, pulmonary to systemic blood flow ratio (Qp/Qs), and Fick-derived cardiac index and PVR were assessed using a 5 Fr or 6 Fr balloon wedge end-hole catheter under fluoroscopic guidance. The catheterization was performed in multiple conditions: in 21% FiO_2_, with 100% FiO_2_, and with 100% FiO_2_ plus 40 ppm inhaled nitric oxide (INOmax; Mallinckrodt). VO_2_, body surface area, and other assumptions by weight and age for ovine models were based on previously published studies in lambs (49–52). Right pulmonary artery angiography was performed with hand injection of radiopaque contrast (Omnipaque 350; GE Healthcare) under fluoroscopy (2005 Powermobil; Siemens), using techniques as described for clinical practice (53, 54). At the conclusion of the cardiac catheterization, the catheters and percutaneous sheath were removed, manual pressure was held until hemostasis, and the skin was closed with tissue adhesive. The lambs were recovered and monitored until back to baseline activity.

### Histopathology of BMPR2^+/–^ F0 lambs with morphometric analysis.

During terminal study, when anesthetized and supported by mechanical ventilation, baseline lung samples were taken from the right middle lobe and immediately flash frozen in liquid nitrogen, placed in phosphate-buffered saline with 4% paraformaldehyde, or formalin-fixed and paraffin embedded. Special staining and immunostaining were performed on 5-μm sections of representative lung samples from 1 control and 2 *BMPR2^+/–^* lambs. Movat pentachrome stain was performed according to established protocols (55). Immunostaining for MKI67 (RRID:AB_2631211, 1:100; Agilent) was performed on the Ventana BenchMark Ultra after CC1 antigen retrieval. Immunofluorescence for VWF (RRID:AB_2315602, 1:100; Agilent), SMA (RRID:AB_2223500, 1:400; Agilent), and the TUNEL assay (Roche In Situ Cell Death Detection Kit, Fluorescein, Sigma-Aldrich) was carried out following citrate pH 6.0 antigen retrieval and serum block; antibodies were incubated overnight at room temperature. VWF was detected with either donkey anti-rabbit–Alexa Fluor 488 or donkey anti-rabbit–cyanine, and SMA was detected with donkey anti-mouse–cyanine (all 1:1000; Jackson ImmunoResearch). Coverslips were mounted using Vectashield fluorescent mounting medium with DAPI (Vector Laboratories). Images were visualized and captured with a digital camera mounted on a Nikon Eclipse 80i microscope using NIS-Elements Advanced Research Software v6.10.01 (Nikon Instruments Inc.). Colocalization of immunofluorescence for either TUNEL and SMA, or TUNEL and VWF, was used to quantify apoptosis in smooth muscle and endothelial cells, respectively. Adjacent sections each stained for a single antigen, either MKI67 and SMA, or MKI67 and VWF, were used to quantify proliferation in smooth muscle and endothelial cells, respectively.

Morphometric analysis of the pulmonary arteries was performed on VWF/SMA immunofluorescently stained sections imaged with the NIS-Elements Research Software. For each lamb, sections from 2 lung blocks were examined. Arteries were excluded from analysis if they were collapsed, or oriented where the muscle wall or endothelium was incompletely visualized. For arterial classification, at least 5 measurements for small and medium sized arteries were carried out, and 3 measurements for the large arteries, the latter limited by the number of large vessel profiles within representative tissue blocks. The outer diameter (OD) across the smallest diameter was measured for each vessel and used to group small (25–100 μm), medium (101–200 μm), and large (201–500 μm) arteries, as previously described (56). The 2 sides of the medial wall (M1 and M2) were measured based on SMA expression, and 2 sides of endothelium (E1 and E2) were measured based on VWF expression. The percentage of medial thickness was calculated as the following: medial thickness (%) = (M1 + M2)/OD × 100, and percentage of intimal thickness was calculated as intimal thickness (%) = (E1 + E2)/OD × 100.

### Statistics.

Normality and homogeneity of variance were assessed prior to testing. A Mann-Whitney test (Prism 11; GraphPad Software) was used to compare the HDR efficiency of the ssODN in combination with either sgRNA1 or sgRNA2. Statistical analyses of the qPCR expression data were performed in R (version 2024.09.0 Build 375) (57). For comparisons between 2 groups (e.g., Lamb 4 vs. WT), an unpaired 2-tailed Welch’s *t* test was used. Differences in intimal and medial thickness among 3 groups (i.e., control, lamb 5, and lamb 6) were determined using 1-way ANOVA with Tukey’s correction for multiple comparison testing (Prism 11; GraphPad Software). To evaluate endothelial and smooth muscle cell proliferation within each arterial size range among 3 groups, Kruskal-Wallis non-parametric tests were used with Dunn’s correction for multiple comparison testing (Prism 11; GraphPad Software). Results are reported as mean ± SEM, and significance was set at a *P* value of less than 0.05.

### Data availability.

Primary data values are available in the Supporting Data Values file and can also be provided by the corresponding authors upon request.

### Study approval.

All protocols and procedures related to the care and evaluation of the animals in this study were approved by the Institutional Animal Care and Use Committees (IACUC) of UCD and UCSF.

## Author contributions

SAD and ALVE designed and coordinated the studies and wrote and edited the manuscript. ARB, DSF, and TFB performed experimental work to optimize editing. BRM and TUB performed the laparoscopic procedures for embryo transfer. OF performed expression analysis. NW and JFT performed molecular analysis, and wrote and edited the manuscript. JMM performed the echocardiography. HN analyzed the echocardiograms. EKA performed the right heart catheterizations. EGJ performed computed tomography, GHD and OAGV performed lung histology. JRF and EDA designed the studies and edited the manuscript. RH managed all aspects of animal care and their studies, including delivery of the F0 lambs.

## Conflict of interest

The authors have declared that no conflict of interest exists.

## Funding support

This work is the result of NIH funding, in whole or in part, and is subject to the NIH Public Access Policy. Through acceptance of this federal funding, the NIH has been given a right to make the work publicly available in PubMed Central.

- NIH grants R01HL133034 (to SAD) and R01HL61284 (to JRF).
- UCSF Pediatric Heart Center Catalyst Award (to SAD, JRF, and EKA).
- UCSF Academic Senate Committee on Research (to SAD).
- Harik-Han Fund (to SAD).
- American Heart Association Transformational Project Award 24TPA1291292 (to SAD, JRF, and EKA).
- Pulmonary Hypertension Association Innovation in PH Research Award (to SAD and JRF).
- UC Davis Jastro Shields Graduate Research Awards (to ARB, DSF, and OF).

## Supplementary Material

Supplemental data

Unedited blot and gel images

Supplemental video 1

Supplemental video 2

Supporting data values

## Figures and Tables

**Table 1:**
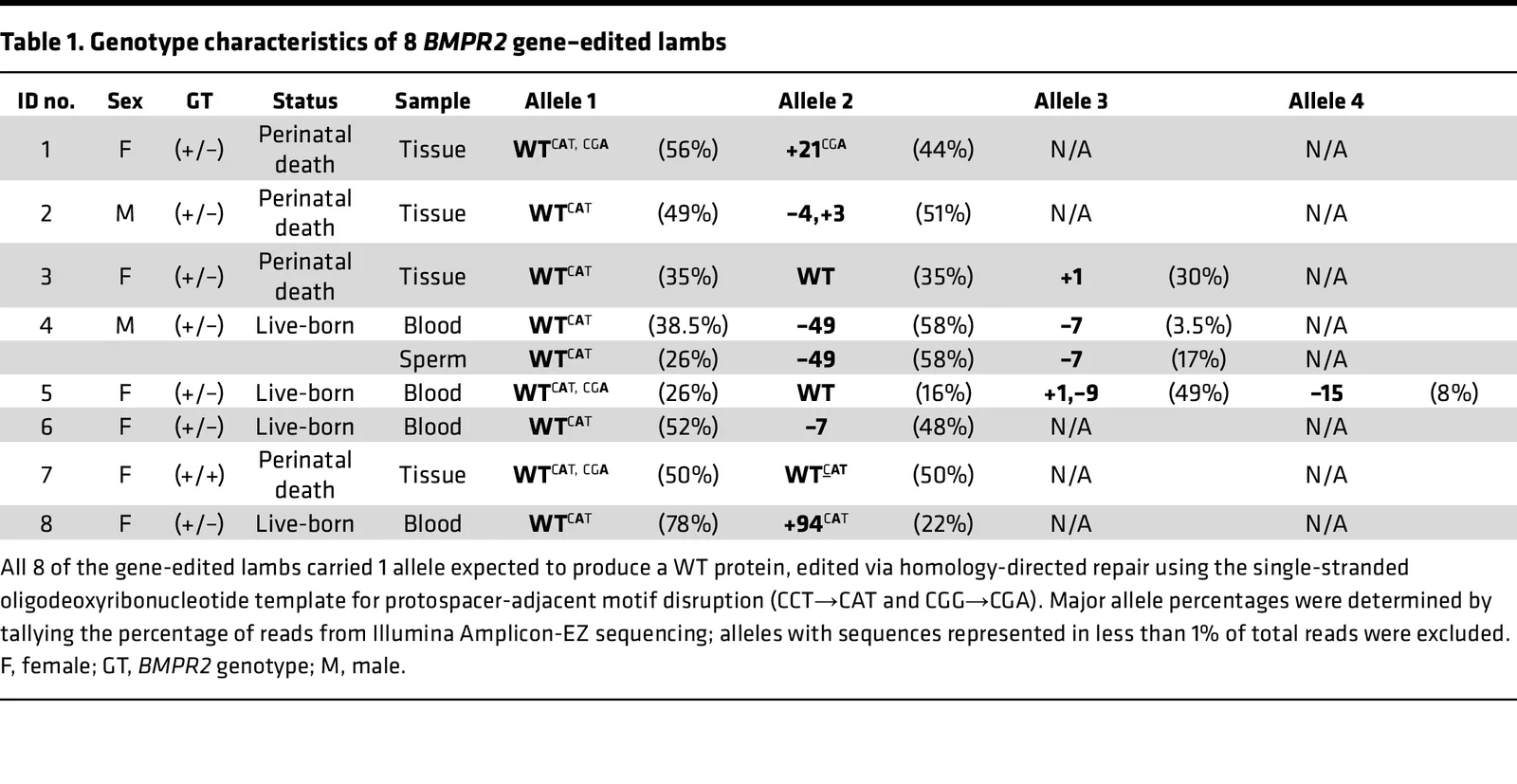
Genotype characteristics of 8 *BMPR2* gene–edited lambs

**Table 2:**
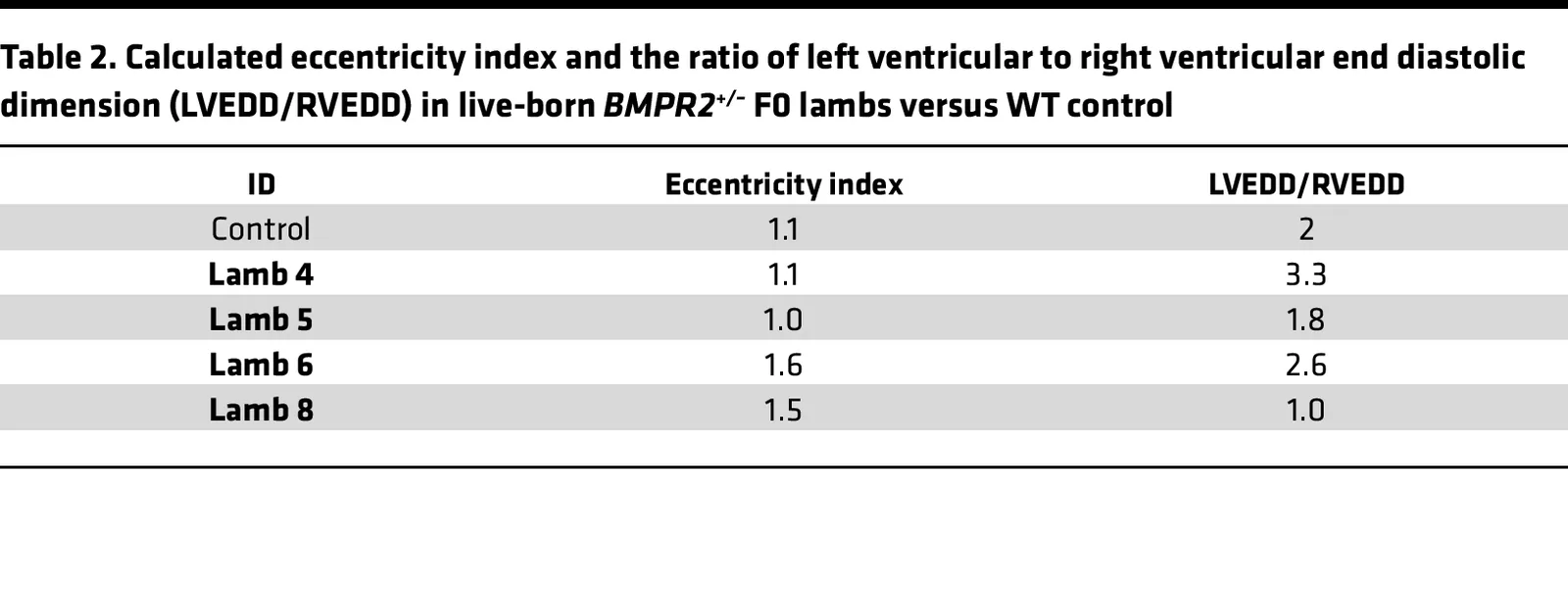
Calculated eccentricity index and the ratio of left ventricular to right ventricular end diastolic dimension (LVEDD/RVEDD) in live-born *BMPR2^+/–^* F0 lambs versus WT control

**Table 3:**
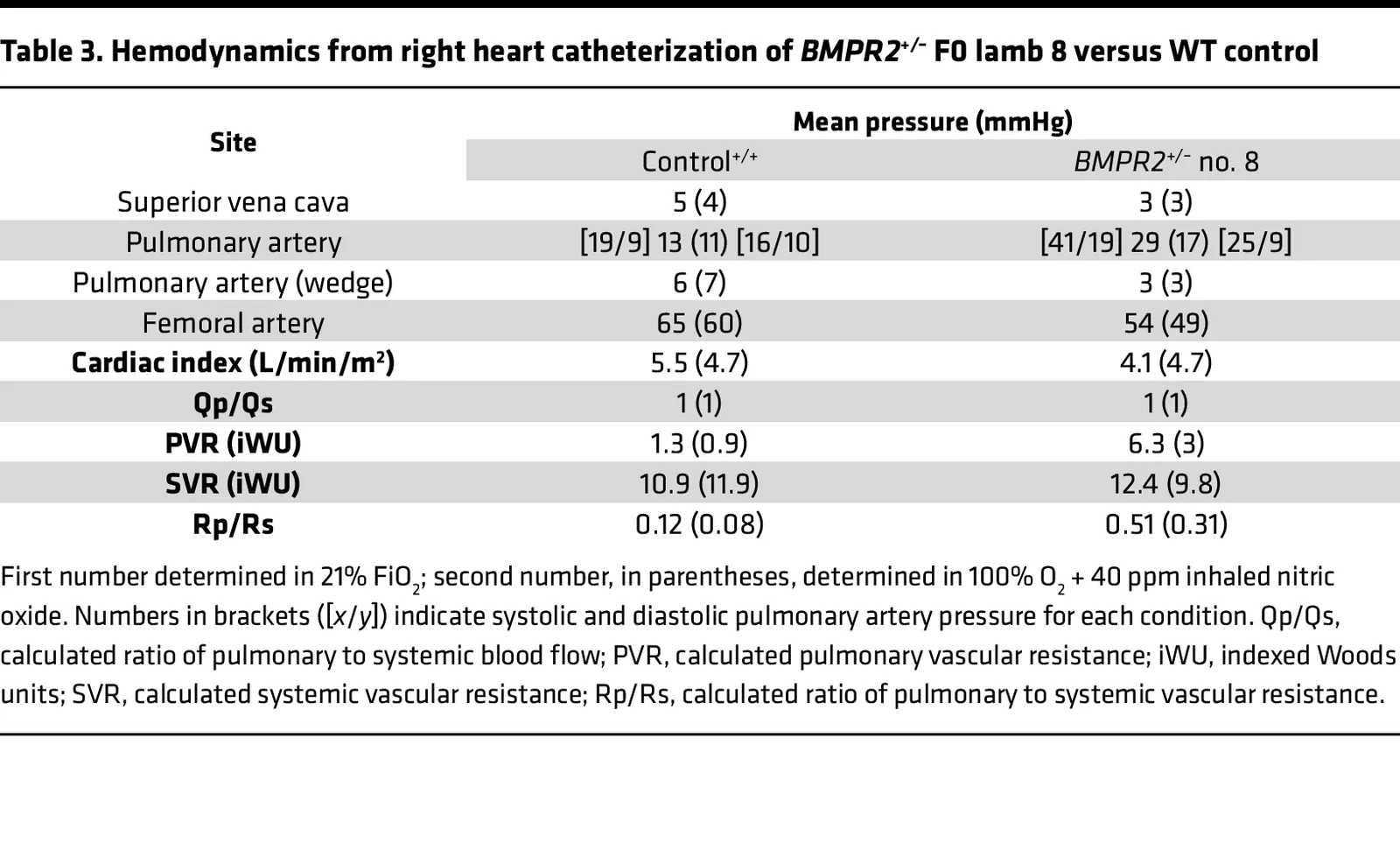
Hemodynamics from right heart catheterization of *BMPR2^+/–^* F0 lamb 8 versus WT control

**Table 4:**
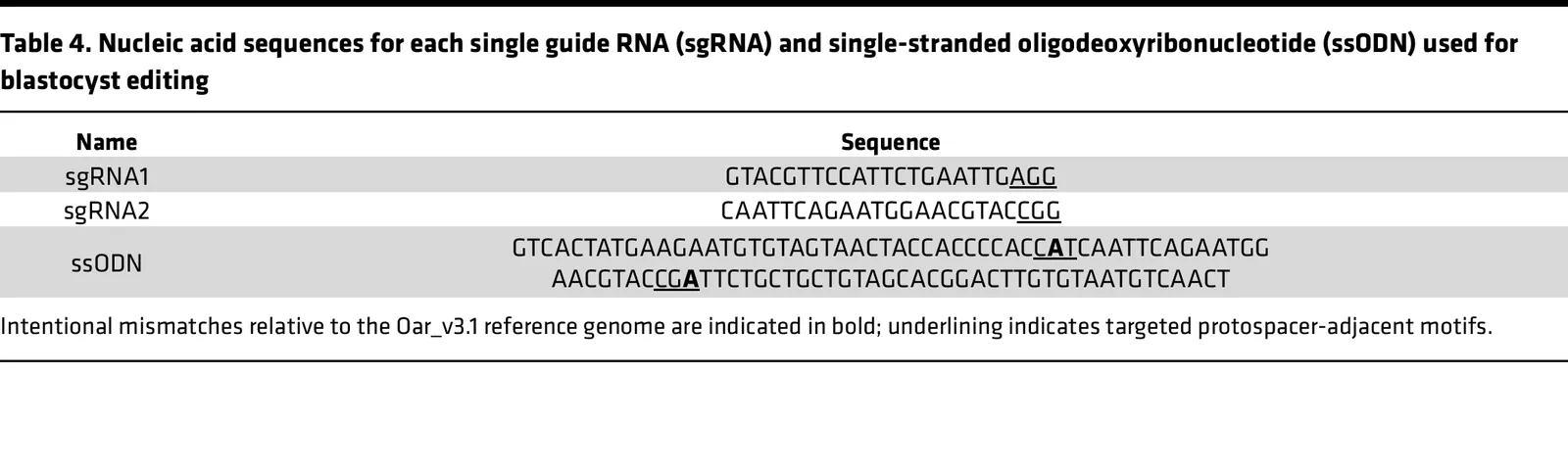
Nucleic acid sequences for each single guide RNA (sgRNA) and single-stranded oligodeoxyribonucleotide (ssODN) used for blastocyst editing

## Version 1 07/14/2026

In-Press Preview

## Version 2 08/24/2026

Electronic publication
