# Supplementary material for: A large animal model of heritable pulmonary arterial hypertension using BMPR2 gene–edited sheep
Source: JCI Insight. 2026 Jul 14;11(16):e205583. doi: 10.1172/jci.insight.205583 (PMC13502180; doi:10.1172/jci.insight.205583)
Supplement: Supplementary file 2 [file jciinsight-11-205583-s166.pdf]

All lanes are used in the final Figure in the manuscript

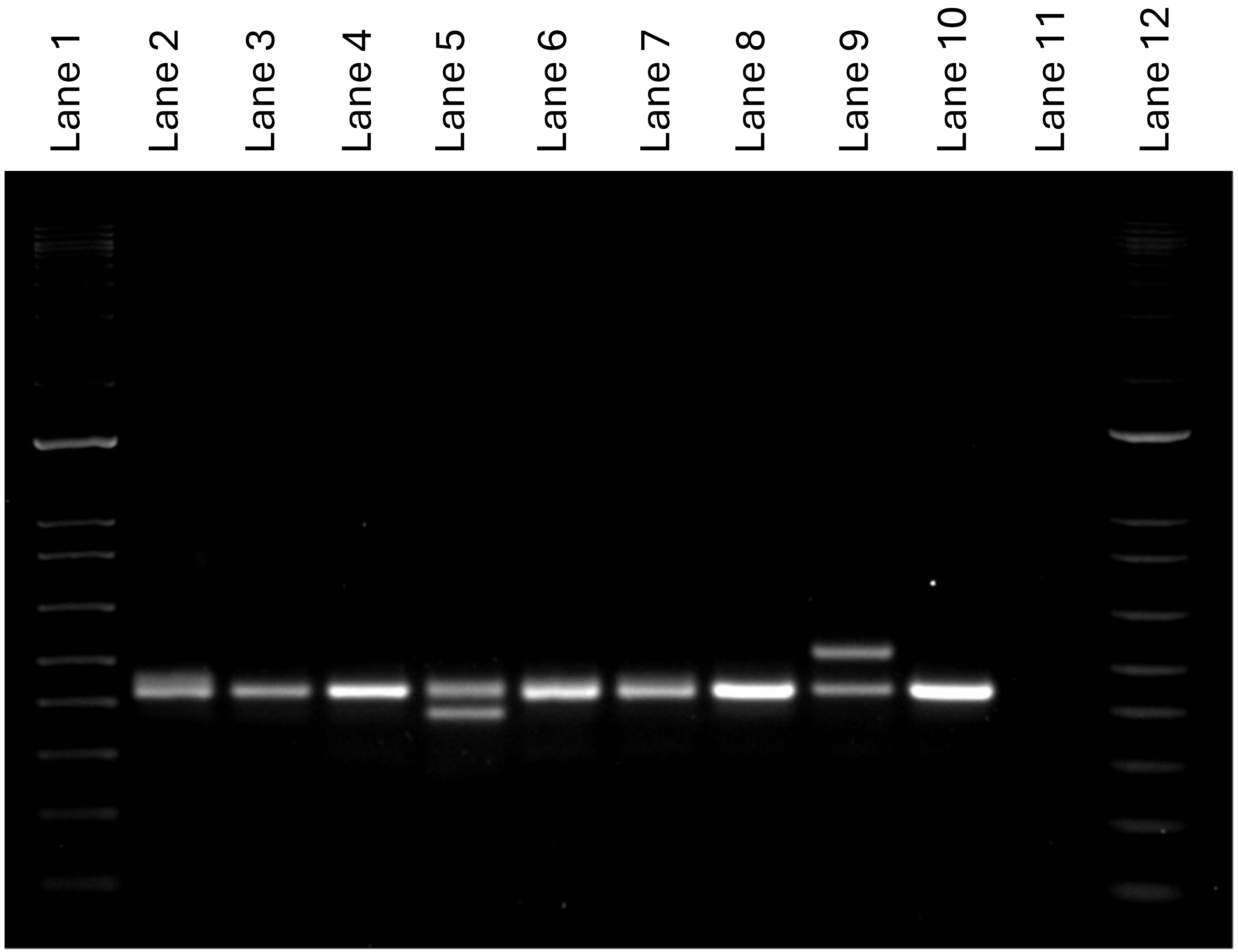

Full unedited gel for Figure 1

All lanes are used in the final Figure in the manuscript

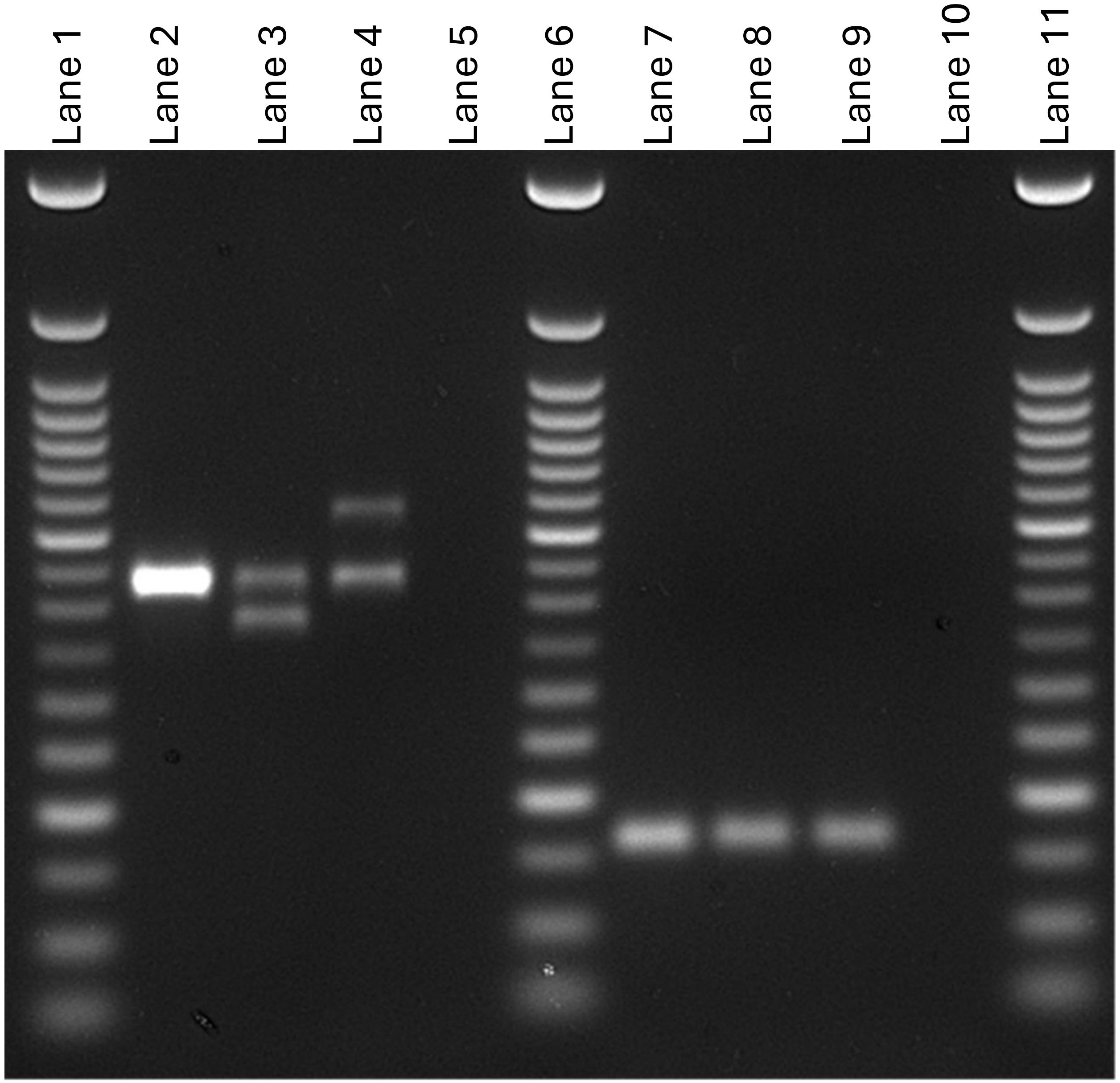

Full unedited gel for Figure 4
